# Association between sleep characteristics and physical functioning in middle-aged and elderly adults: findings from Chinese cohorts

**DOI:** 10.1007/s40520-025-03020-9

**Published:** 2025-04-07

**Authors:** ZhaoLiang Zhang, LieHui Yao

**Affiliations:** The Affiliated YiXing Hospital of Jiangsu University, Yixing, Jiangsu 214200 China

**Keywords:** Cohort study, Physical function, Muscle strength, Sleep quality, Aging

## Abstract

**Aims:**

Aging is associated with declines in muscle strength and physical function, and sleep plays a crucial role in maintaining musculoskeletal health. This cohort study, based on the China Health and Retirement Longitudinal Study (CHARLS), aims to explore the relationship between sleep characteristics and physical performance in middle-aged and elderly individuals.

**Methods:**

A total of 2,998 participants from the CHARLS cohort were included, with baseline data from the 2011 survey and follow-up data from the 2015 survey. Nighttime sleep and napping patterns were assessed, and changes in sleep duration over time were categorized. Physical functioning was assessed through handgrip strength, the chair stand test, the walking test, appendicular skeletal muscle mass, and SPPB scores. Multiple linear regression and restricted cubic spline models were employed to analyze the effects of sleep patterns on muscle health.

**Results:**

After adjusting for all confounding factors, multifactor liner regression indicated that napping durations exceeding 90 min were associated with reduced grip strength (*β* = -0.39, 95% CI: -1.43, -0.01, *P* < 0.05) and longer chair stand test times (*β* = 0.59, 95% CI: 0.18, 0.99, *P* < 0.01). The restricted cubic spline indicated a clear U-shaped relationship between nap duration and grip strength (non-linear *P* < 0.05), whereas no such relationship was observed between nap duration and performance on the chair stand test. In subgroup analyses addressing significant confounding factors, it was found that participants older than 65 years, males, and those with low levels of daily activity exhibited greater sensitivity to the effects of sleep on physical function. The joint analysis showed that compared to individuals with less than 6 h of nighttime sleep and no napping, those with 6–8 h of nighttime sleep and 30–90 min of napping exhibited longer chair stand test durations (*β* = 0.63, 95% CI: 0.06, 1.20, *P* < 0.05). Additionally, individuals with 6–8 h of nighttime sleep and naps exceeding 90 min showed longer walking times (*β* = 1.44, 95% CI: 0.68, 2.21, *P* < 0.01).

**Conclusions:**

Both insufficient and excessive nighttime sleep at baseline were linked to diminished physical performance. For older adults, a nap duration approximately 50 min may be potentially optimal for maintaining grip strength. These findings highlight the importance of proper sleep management, particularly for men over 65 years old with low activity levels, in preserving physical function and reducing the risk of mobility decline.

**Supplementary Information:**

The online version contains supplementary material available at 10.1007/s40520-025-03020-9.

## Background

The aging population is becoming prominent demographic trend worldwide, with the United Nations General Assembly declaring 2021–2030 as the “Decade of Healthy Ageing” [1]. The demographic shift brought by aging populations has placed significant pressure on socio-economic systems. The growing number of elderly individuals not only burdens the working-age population but also strains public finances. A decline in physical functioning, particularly in terms of loss of muscle mass, is a prominent aspect of aging. As people age, physical decline tends to accelerate and is often linked to adverse health outcomes, such as depression, falls, and disabilities [2]. These outcomes present an escalating challenge for healthcare systems as the aging process accelerates.

As the aging process accelerates, maintaining good health becomes more challenging, and various factors, including lifestyle habits, play a significant role in mitigating or exacerbating age-related declines. Among these factors, sleep is a critical element that influences both physical and mental well-being, particularly in middle-aged and elderly individuals. The mechanisms underlying the relationship between sleep and aging-related physical decline remain complex and multifaceted. One potential pathway involves sleep’s crucial role in muscle repair and growth. During deep sleep, the body secretes growth hormone, which is essential for muscle protein synthesis and the maintenance of muscle mass [3]. Besides, Chronic sleep disturbances have been linked to elevated levels of pro-inflammatory cytokines, such as interleukin-6 and tumor necrosis factor-alpha, which contribute to muscle degradation [4]. Given these physiological connections, understanding the optimal sleep patterns for maintaining muscle health and physical function is crucial for promoting healthy aging.

Research has suggested that sleep duration among middle-aged and elderly individuals is linked with various health issues, with appropriate sleep being essential for the regulation of metabolic and physiological functions. In older individuals, Inadequate or disrupted sleep patterns, such as shorter sleep duration, prolonged sleep onset latency, and fragmented sleep, are commonly observed and are associated with several age-related diseases [5–7]. However, the relationship between sleep duration and physical functioning in older adults remains inconsistent. Some meta-analyses suggested a U-shaped relationship between sleep duration and sarcopenia, indicating that both insufficient and excessive sleep might have negative impacts on physical function [8, 9]. For example, Fu L et al. [10] found only a negative correlation between shorter sleep duration and decreased muscle mass in elderly Chinese community members, while Shibuki et al. [11] reported that individuals with longer sleep durations were more likely to develop sarcopenia or reduced physical function in a Japanese cohort. In addition to nighttime sleep, napping is another prevalent sleep habit, particularly in East Asian countries. It has been reported that prolonged naps are linked to cognitive decline and impaired fasting glucose [12, 13]. However, there is limited research exploring the impact of nap duration on physical functioning, and further investigation is needed to understand this relationship.

Despite the growing study on sleep and physical function in older individuals, several important gaps remain. Most existing studies rely on cross-sectional designs, which limit the ability to establish causal relationships between sleep characteristics and physical function. Moreover, many studies lack clear and consistent classification criteria for different sleep durations, making comparisons across studies challenging. While considerable attention has been paid to the effects of insufficient sleep, the potential adverse effects of excessive sleep have not been adequately explored. Additionally, there is a lack of research on the combined effects of nighttime sleep and napping on physical function. Most studies have focused on either nighttime sleep or nap duration in isolation, neglecting the cumulative impact that both may have on overall health. Given the rapid aging of the population and the increasing prevalence of sleep-related concerns in modern society, this research is particularly timely and important.

To better understand these issues, our study leverages data from the China Health and Retirement Longitudinal Study (CHARLS), a large-scale longitudinal survey designed to study the health, socioeconomic status, family, and community environment of middle-aged and elderly individuals in China. Organized and implemented by the National School of Development at Peking University, CHARLS aims to provide valuable data to policymakers and researchers on China’s aging population. The survey employs a multi-stage sampling method proportional to population size, covering 150 county-level units, 450 village-level units, and approximately 17,000 individuals from 10,000 households [14]. The project has received approval from the Biomedical Ethics Review Committee of Peking University, and all participants have provided informed consent [5]. By utilizing the comprehensive data collected in CHARLS, this study aims to explore the relationship between sleep patterns and physical functioning in middle-aged and elderly individuals, offering critical insights into healthy aging strategies. Our study aims to address these gaps and provide a more comprehensive understanding of how different sleep patterns—including both nighttime sleep and napping—affect physical functioning in middle-aged and elderly individuals. The findings of this study may offer valuable insights into the role of circadian rhythms in maintaining physical function and promoting healthy aging.

## Methods

### Study population

**Fig. 1:**
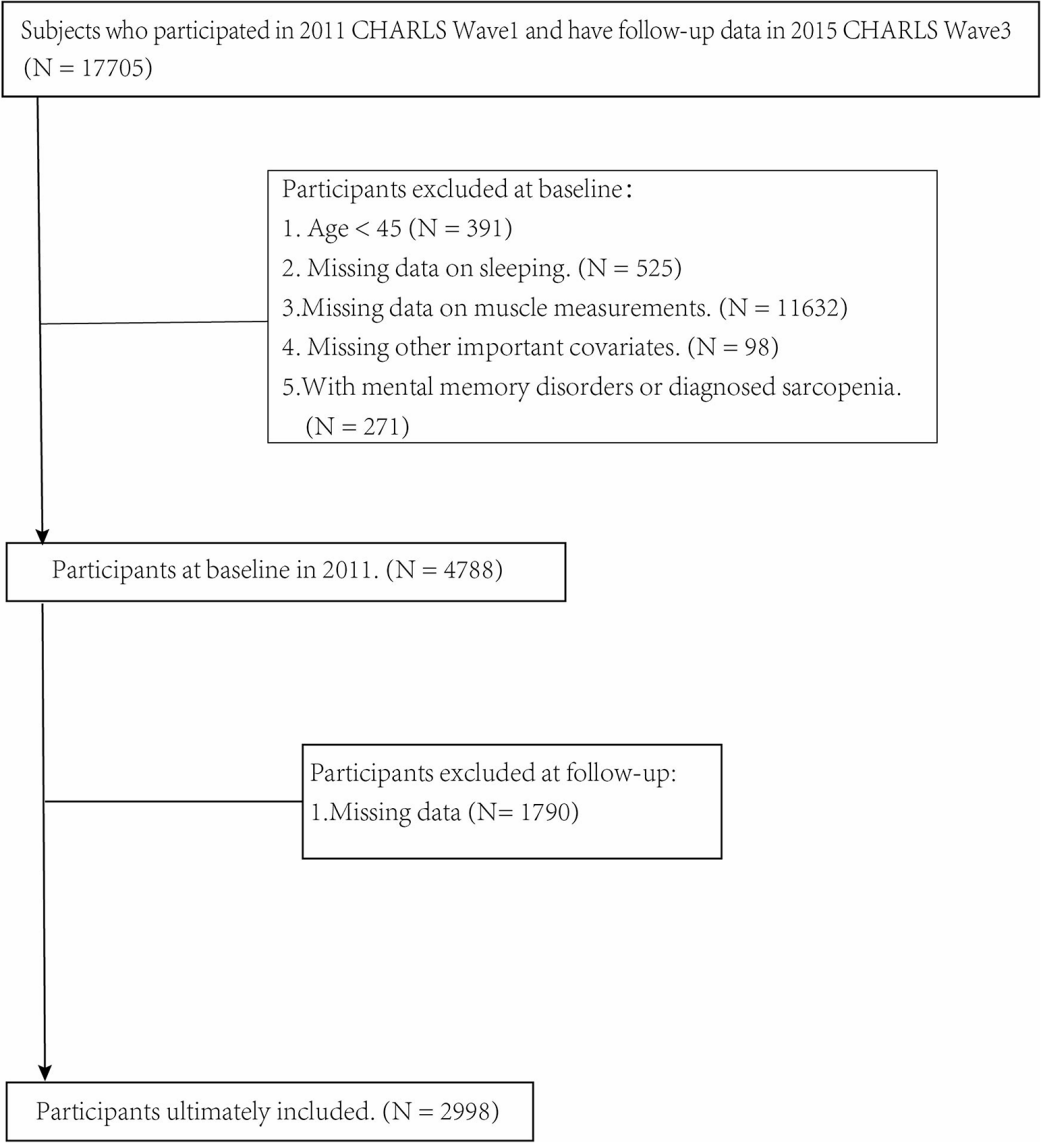
Flowchart of sample selection process

The study population was drawn from 2011 to 2015 of CHARLS. Participants were excluded based on the following criteria: (1) age < 45 years; (2) missing information on sleep duration or napping duration; (3) inability to complete or missing information in physical function tests; (4) diagnosed with sarcopenia at baseline (2019 criteria of the Asian Working Group for Sarcopenia (AWGS)) [13]; (5) presence of mental or memory-related diseases. Ultimately, this analysis included 2,998 participants (Fig. 1).

### Physical functioning

Physical function was assessed using handgrip strength tests, chair stand tests, walking tests, appendicular skeletal muscle mass (ASM) measurements, and Short Physical Performance Battery (SPPB) scores. Handgrip strength was measured using a dynamometer, with the average of two trials recorded as the final result. Chair stand and walking tests were conducted to evaluate lower limb strength and mobility, while ASM was calculated using a validated formula, adjusted for height (ASM/HT²) to assess muscle mass [14]. The SPPB score, which includes standing balance, walking speed, and a repeated chair stand test, was used to provide a comprehensive evaluation of participants’ physical function [15]. Detailed measurement procedures as well as SPPB score are provided in the supplementary material.

### Sleeping duration

The duration of sleep primarily relies on participants’ subjective responses from the questionnaire. Questions include: “In the past month, how many hours did you actually fall asleep each night on average? (likely shorter than the time spent lying in bed)” and “During the past month, how long did you take a nap after lunch?“. Nighttime sleep duration is divided into 3 groups: <6 h, 6–8 h, and > 8 h, based on previous studies that have identified these thresholds as relevant for assessing the association between sleep duration and health outcomes in middle-aged and elderly populations [18]. The 6–8 h range is always considered optimal for maintaining overall health in this demographic. Napping duration is categorized into 4 groups: No napping, < 30 min, 30–90 min, and > 90 min. Based on changes in nighttime sleep duration from baseline to follow-up, participants are divided into 3 groups: Not change, increased, and decreased.

### Covariates

We included additional sociodemographic and health-related covariates in the analysis [5, 16]. Sociodemographic characteristics comprised age, gender, residence area (rural or urban), education level (secondary education or above vs. below), marital status (married/cohabitation vs. other), and insurance coverage. Health-related characteristics included BMI, smoking status, alcohol consumption, the presence of 14 common comorbidities, and daily activity scores.

Age, marital status, insurance coverage, and education level were obtained from the corresponding questionnaire modules. Alcohol consumption was defined as a history of habitual intake of alcoholic beverages including spirituous liquor, wine, and rice wine, either in the past or present. Smoking status was defined as a history of habitual use of cigarettes, pipe tobacco, dry tobacco, or chewing tobacco, either in the past or present. BMI was calculated based on the participants’ height and weight, which were measured on-site. Presence of comorbidities and daily activity scores were obtained from retrospective questionnaires. Detailed explanations of this section are provided in the supplementary materials.

### Statistical analysis

Continuous variables were described using mean and standard deviation (M, SD), while categorical variables were described using frequency and percentage (n, %). Multivariate linear regression models were constructed to describe the relationship between different sleep characteristics and physical functioning. Nighttime sleep of 6–8 h, no napping, and no change in nighttime duration from baseline to follow-up were used as the reference group. To examine the relationship between different sleep characteristics and physical functioning, we adopted a stepwise approach by progressively adding covariates to the models. Model 1 included only 3 sleep characteristics. Model 2 included age, marital status, insurance coverage, and education, which serve as basic sociodemographic factors. Model 3 further adjusted for lifestyle factors: alcohol consumption, smoking status, BMI, number of comorbidities, and daily activity levels. Model 4 included all covariates to provide the most comprehensive analysis, controlling for a wide range of factors that might influence the relationship between sleep and physical functioning. Restricted cubic splines (RCS) were used to model the dose-response relationship of continuous variables. This method allows for the flexible modeling of nonlinear relationships by dividing the continuous variable into intervals based on data percentiles and fitting piecewise cubic splines to these intervals. RCS can provide a more accurate representation of complex relationships between continuous predictors and outcomes, as opposed to assuming a linear association. A *P*-value of less than 0.05 was considered statistically significant. The significant codes were as follows: “***” for *P* < 0.001, “**” for *P* < 0.01, and “*” for *P* < 0.05. All statistical analyses were conducted using R version 4.4.1.

## Results

### Participant characteristics

In total, 2,959 middle-aged and elderly individuals were included in the study, comprising 1,362 females and 1,636 males. Participants with memory-related diseases were excluded from the study, as most variables were assessed based on retrospective questionnaires. Individuals with memory impairment may introduce substantial recall bias, which could affect the accuracy of results. Besides, they could also impact physical function and sleep. The average age was 66.31 ± 5.34 years. There were statistically significant differences between genders in all variables except for the number of chronic diseases, unless otherwise specified by tumorous diseases. As for sleeping patterns, males tended to sleep slightly longer at night (6.56 h) compared to females (6.02 h). Males napped for a longer duration (46.39 min) compared to females (36.09 min). A higher proportion of females napped for < 30 min (7.6%) compared to males (5.9%), and more males napped for > 90 min (18.1%) compared to females (12.0%). Specific basic characteristics are shown in Table 1.

**Table 1:** Baseline characteristics of participants

	Overall	Female		Male		*P*-values	
n	2998	1362	1636				
Age (years, mean (SD))	66.31 (5.34)	65.90 (5.20)	66.65 (5.43)		< 0.001		
Education = Secondary education or above (n, %)	43 (1.4)	9 (0.7)	34 (2.1)		0.002		
Residence = Urban (n, %)	470 (15.7)	240 (17.6)	230 (14.1)		0.009		
Marital status = Married/cohabitation (n, %)	2525 (84.2)	1073 (78.8)	1452 (88.8)		< 0.001		
Under Insurance (n, %)	2850 (95.1)	1291 (94.8)	1559 (95.3)		0.581		
Number of chronic diseases (n, %)					0.003		
No self-reported chronic diseases	797 (26.6)	325 (23.9)	472 (28.9)				
= 1	898 (30.0)	406 (29.8)	492 (30.1)				
= 2	641 (21.4)	296 (21.7)	345 (21.1)				
>= 3	662 (22.1)	335 (24.6)	327 (20.0)				
History of drinking (n, %)	1000 (33.4)	162 (11.9)	838 (51.2)		< 0.001		
History of smoking (n, %)	1361 (45.4)	145 (10.6)	1216 (74.3)		< 0.001		
BMI (mean (SD))	23.29 (3.86)	24.04 (4.05)	22.66 (3.57)		< 0.001		
Hypertension (n, %)	897 (30.0)	462 (34.0)	435 (26.7)		< 0.001		
Diabetes or high blood sugar (n, %)	186 (6.2)	104 (7.7)	82 (5.1)		0.004		
Cancer or malignant tumor (n, %)	25 (0.8)	14 (1.0)	11 (0.7)		0.388		
Heart disease (n, %)	424 (14.2)	217 (16.0)	207 (12.7)		0.013		
Arthritis or rheumatism (n, %)	1124 (37.5)	589 (43.3)	535 (32.8)		< 0.001		
Asthma (n, %)	152 (5.1)	48 (3.5)	104 (6.4)		0.001		
Daily activity level (n, %)					< 0.001		
Low	1257 (41.9)	418 (30.7)	839 (51.3)				
Medium	863 (28.8)	402 (29.5)	461 (28.2)				
High	878 (29.3)	542 (39.8)	336 (20.5)				
Nighttime (hours, mean (SD))	6.32 (2.10)	6.02 (2.23)	6.56 (1.95)		< 0.001		
Napping time (minutes, mean (SD))	41.71 (46.34)	36.09 (43.81)	46.39 (47.86)		< 0.001		
Napping duration (n, %)					< 0.001		
< 30 min	200 (6.7)	104 (7.6)	96 (5.9)				
> 90 min	460 (15.3)	164 (12.0)	296 (18.1)				
30–90 min	1149 (38.3)	509 (37.4)	640 (39.1)				
No napping	1189 (39.7)	585 (43.0)	604 (36.9)				
Variations in nighttime sleep duration (n, %)					< 0.001		
Increased	1179 (39.3)	520 (38.2)	659 (40.3)				
Not changed	708 (23.6)	268 (19.7)	440 (26.9)				
Decreased	1111 (37.1)	574 (42.1)	537 (32.8)				
Nighttime sleep duration (n, %)					< 0.001		
< 6 h	1000 (33.4)	554 (40.7)	446 (27.3)				
> 8 h	334 (11.1)	142 (10.4)	192 (11.7)				
6–8 h	1664 (55.5)	666 (48.9)	998 (61.0)				
Handgrip (kg, mean (SD))	27.74 (8.83)	22.05 (6.29)	32.47 (7.78)		< 0.001		
Pace of walking (seconds, mean (SD))	3.62 (3.89)	3.94 (5.58)	3.36 (1.25)		< 0.001		
Chair stand test(seconds, mean (SD))	10.40 (3.99)	10.95 (4.48)	9.94 (3.47)		< 0.001		
ASM/HT^2^ (kg/m^2^, mean (SD))	8.86 (1.77)	10.60 (0.84)	7.41 (0.75)		< 0.001		
SPPB score (n, %)					< 0.001		
4	4 (0.1)	3 (0.2)	1 (0.1)				
5	35 (1.2)	18 (1.3)	17 (1.0)				
6	350 (11.7)	212 (15.6)	138 (8.4)				
7	116 (3.9)	75 (5.5)	41 (2.5)				
8	170 (5.7)	98 (7.2)	72 (4.4)				
9	1452 (48.4)	567 (41.6)	885 (54.1)				
10	189 (6.3)	99 (7.3)	90 (5.5)				
11	404 (13.5)	187 (13.7)	217 (13.3)				
12	278 (9.3)	103 (7.6)	175 (10.7)				

### Relationships between sleep characteristics and physical functioning

Multivariate linear regression revealed that, after adjusting for confounding factors in Model 2 and Model 3, both short nighttime sleep duration (< 6 h) and prolonged nighttime sleep were negatively associated with handgrip strength. Conversely, napping for 30–90 min appeared to mitigate grip strength decline. Additionally, shorter nighttime sleep, excessive napping (> 90 min), and changes in nighttime sleep duration (either an increase or decrease) were associated with poorer chair stand test performance and higher ASM/HT². A decrease in nighttime sleep duration was also linked to lower SPPB scores.

After adjusting for all covariates in Model 4, we found that extended nighttime sleep duration was associated with lower grip strength (*β* = -0.61, 95% CI: -1.23, -0.01, *P* < 0.05) and longer chair stand test completion time (*β* = 0.40, 95% CI: 0.04, 0.75, *P* < 0.05). Additionally, napping for more than 90 min was linked to reduced grip strength (*β* = -0.39, 95% CI: -1.43, -0.01, *P* < 0.05) and poorer chair stand test performance (*β* = 0.59, 95% CI: 0.18, 0.99, *P* < 0.01). This suggests maintaining a moderate and stable sleep pattern may be beneficial for physical function. Detailed regression results are presented in Table 2.

**Table 2:** Multivariate linear regression results of sleep characteristics and physical functioning

		Handgrip (β-Coefficients (95%CI))				ASM/HT^2^ (β-Coefficients (95%CI))				Chair stand test (β-Coefficients (95%CI))				Pace of walking (β-Coefficients (95%CI))				SPPB score (β-Coefficients (95%CI))			
	Case/%	Model 1	Model 2	Model 3	Model 4	Model 1	Model 2	Model 3	Model 4	Model 1	Model 2	Model 3	Model 4	Model 1	Model 2	Model 3	Model 4	Model 1	Model 2	Model 3	Model 4
**Nighttime sleep duration**																					
6–8 h	1664/55.50%	Ref	Ref	Ref	Ref	Ref	Ref	Ref	Ref	Ref	Ref	Ref	Ref	Ref	Ref	Ref	Ref	Ref	Ref	Ref	Ref
<6 h	1000/33.36%	-2.35(-3.04, -1.67)***	-2.12(-2.80, -1.45)***	-0.88(-1.47, -0.29)**	-0.10(-0.63, 0.42)	0.44(0.30, 0.58)***	0.45(0.32, 0.59)***	0.22(0.14, 0.31)***	-0.01(-0.02, 0.02)	0.60(0.28, 0.91)***	0.53(0.22, 0.84)***	0.17(-0.13, 0.46)	0.11(-0.18, 0.41)	0.20(-0.11, 0.50)	0.17(-0.13, 0.48)	0.02(-0.29, 0.33)	-0.01(-0.32, 0.30)	-0.12(-0.25, 0.02)	-0.12(-0.25, 0.02)	-0.05(-0.19, 0.08)	-0.02(-0.16, 0.12)
>8 h	334/11.14%	-1.31(-2.34, -0.28)*	-0.84(-1.85, 0.17)	-0.80(-1.68, 0.08)	-0.44(-1.21, 0.34)	-0.03(-0.23, 0.18)	0.04(-0.16, 0.24)	0.09(-0.03, 0.21)	-0.01(-0.05, 0.02)	0.43(-0.04, 0.90)	0.26(-0.21, 0.72)	0.23(-0.21, 0.67)	0.20(-0.24, 0.64)	0.71(0.25, 1.17)**	0.64(0.19, 1.10)**	0.63(0.18, 1.08)**	0.62(0.16, 1.07)	-0.18(-0.383, 0.02)	-0.18(-0.38, 0.02)	-0.18(-0.38, 0.02)	-0.17(-0.37, 0.03)
**Napping duration**																					
No napping	1189/39.66%	Ref	Ref	Ref	Ref	Ref	Ref	Ref	Ref	Ref	Ref	Ref	Ref	Ref	Ref	Ref	Ref	Ref	Ref	Ref	Ref
< 30 min	200/6.67%	0.75(-0.57, 2.07)	0.82(-0.46, 2.11)	0.66(-0.46, 1.78)	0.88(-0.10, 1.86)	0.14(-0.13, 0.40)	0.11(-0.15, 0.37)	0.05(-0.11, 0.21)	-0.01(-0.05, 0.03)	0.19(-0.41, 0.80)	0.16(-0.42, 0.75)	0.14(-0.42, 0.70)	0.13(-0.43, 0.68)	-0.18(-0.76, 0.40)	-0.19(-0.77, 0.40)	-0.16(-0.74, 0.41)	-0.17(-0.75, 0.41)	0.13(-0.13, 0.38)	0.13(-0.12, 0.39)	0.13(-0.12, 0.39)	0.14(-0.11, 0.40)
30–90 min	460/15.34%	1.21(0.50, 1.93)***	1.06(0.36, 1.76)**	0.83(0.22, 1.44)**	-0.72(-0.15, 0.93)	-0.01(-0.15, 0.13)	-0.01(-0.15, 0.13)	-0.12(-0.21, -0.04)**	0.00(-0.02, 0.03)	0.18(-0.14, 0.51)	0.22(-0.10, 0.54)	0.23(-0.08, 0.53)	0.26(-0.04, 0.57)	-0.21(-0.53, 0.10)	-0.20(-0.52, 0.12)	-0.17(-0.48, 0.15)	-0.15(-0.46, 0.17	0.09(-0.05, 0.23)	0.10(-0.04, 0.24)	0.10(-0.04, 0.23)	0.08(-0.06, 0.22)
> 90 min	1149/38.33%	0.65(-0.30, 1.60)	0.77(-0.16, 1.69)	0.19(-0.61, 0.99)	-0.39(-1.43, -0.01)*	-0.36(-0.55, -0.17)***	-0.34(-0.53, -0.16)***	-0.25(-0.37, -0.14)***	0.01(-0.02, 0.04)	0.53(0.10, 0.96)*	0.48(0.06, 0.90)*	0.52(0.11, 0.92)*	0.59(0.18, 0.99)**	-0.15(-0.57, 0.27)	-0.17(-0.59, 0.25)	-0.13(-0.54, 0.29)	-0.09(-0.51, 0.33)	-0.01(-0.20, 0.17)	-0.01(-0.19, 0.18)	-0.02(-0.20, 0.16)	-0.06(-0.24, 0.13)
**Variations in nighttime sleep duration**																					
Not changed	708/23.62%	Ref	Ref	Ref	Ref	Ref	Ref	Ref	Ref	Ref	Ref	Ref	Ref	Ref	Ref	Ref	Ref	Ref	Ref	Ref	Ref
Increased	1179/39.33%	-1.54(-2.36, -0.72)***	-1.40(-2.20, -0.60)***	-0.92(-1.62, -0.22)**	-0.61(-1.23, -0.01)*	0.19(0.02, 0.35)*	0.22(0.06, 0.38)**	0.09(-0.01, 0.19)	0.00(-0.02, 0.03)	0.47(0.10, 0.84)*	0.41(0.05, 0.78)*	0.31(-0.03, 0.66)	0.29(-0.05, 0.64)	0.27(-0.09, 0.64)	0.25(-0.11, 0.61)	0.21(-0.15, 0.57)	0.20(-0.16, 0.56)	-0.14(-0.30, 0.02)	-0.14(-0.30, 0.02)	-0.12(-0.28, 0.04)	-0.11(-0.27, 0.05)
Decreased	1111/37.06%	-1.70(-2.53, -0.87)***	-1.61(-2.42, -0.80)***	-0.62(-1.32, 0.09)	0.08(-0.55, 0.70)	0.45(0.28, 0.61)***	0.44(0.28, 0.60)***	0.19(0.07, 0.27)***	-0.01(-0.04, 0.01)	0.75(0.37, 1.13)***	0.74(0.37, 1.11)***	0.44(0.09, 0.80)*	0.40(0.04, 0.75)*	-0.00(-0.37, 0.37)	-0.01(-0.38, 0.35)	-0.14(-0.51, 0.22)	-0.17(-0.54, 0.20)	-0.20(-0.36, -0.04)*	-0.20(-0.36, -0.04)*	-0.15(-0.31, -0.01)*	-0.12(-0.28, 0.04)

Model 1: Crude model

Model 2: Age, marital status, insurance coverage, education

Model 3: Age, marital status, insurance coverage, education, alcohol consumption, smoking status, BMI, number of comorbidities, daily activity level

Model 4: Age, marital status, insurance coverage, education, alcohol consumption, smoking status, BMI, number of comorbidities, daily activity level, gender

**Fig. 2:**
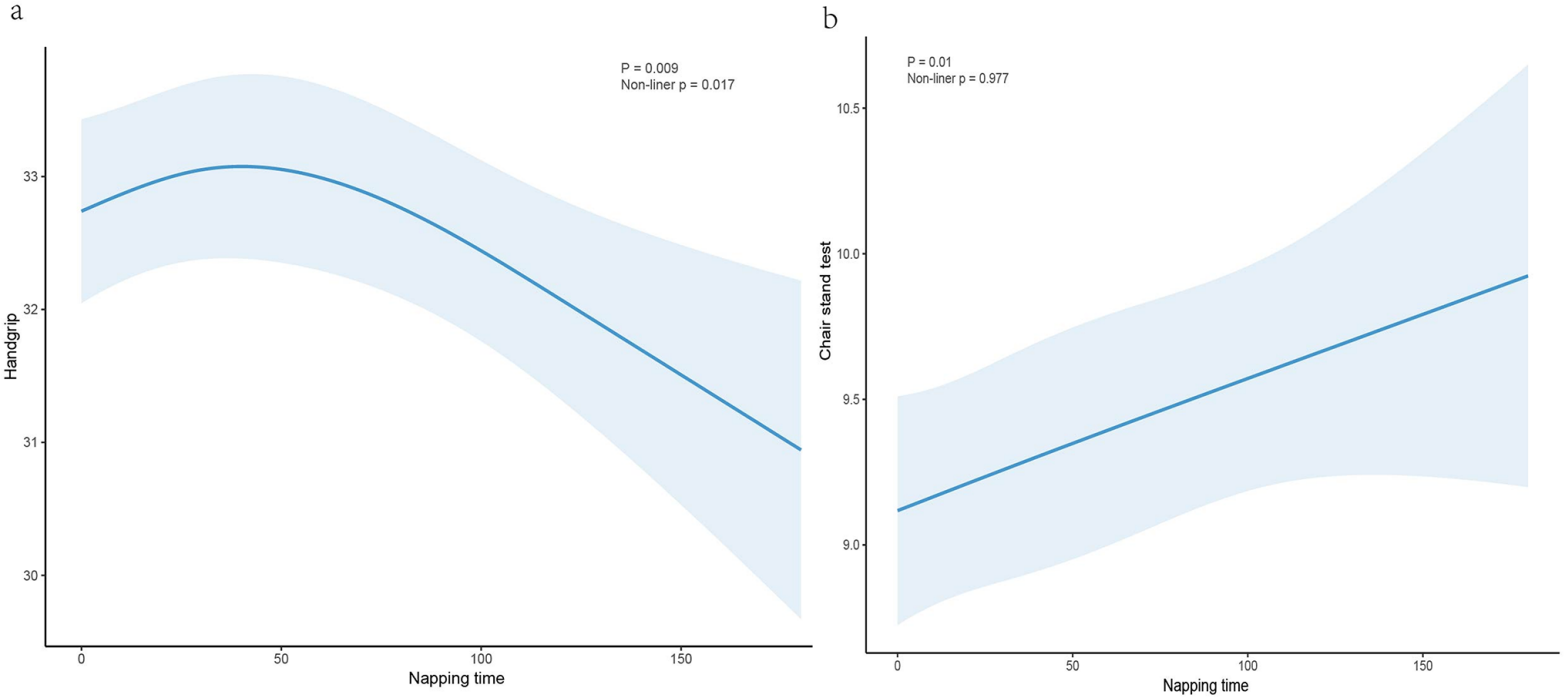
Dose-response relationship between nap duration and grip strength, and chair stand test time

To further investigate the dose-response relationship between napping duration and physical function, we treated napping duration as a continuous variable in Model 4. As shown in Fig. 2, the dose-response relationship between napping duration and handgrip strength in middle-aged and elderly individuals reveals a U-shaped association (non-linear *P* < 0.05). Specifically, grip strength tends to decline significantly if napping duration exceeds 50 min. However, no U-shaped relationship was observed between napping duration and chair stand test performance.

### Differences in the impact of sleep characteristics on physical functioning by daily activity

To control for confounding factors, we selected gender, age, and daily activity levels as the primary variables for subgroup analysis. The results indicated that the impact of sleep characteristics on physical function varied across the 3 levels of daily activity (Table 3). Among middle-aged and elderly individuals with low daily activity levels, decreased nighttime sleep duration was significantly associated with lower handgrip strength (*β* = -1.18, 95% CI: -2.15, -0.21, *P* < 0.01) and longer chair stand test time (*β* = 0.41, 95% CI: 0.05, 0.77, *P* < 0.05).Additionally, prolonged nighttime sleep (> 8 h) was associated with longer chair stand test time (*β* = 0.60, 95% CI: 0.11, 1.09, *P* < 0.05), longer walking time (*β* = 1.30, 95% CI: 0.37, 2.23, *P* < 0.001), and lower SPPB scores (*β* = -0.31, 95% CI: -0.59, -0.03, *P* < 0.05). Furthermore, excessive napping (> 90 min) was also found to be associated with a decline in grip strength (*β* = -1.18, 95% CI: -2.25, -0.12, *P* < 0.05) and longer chair stand test time (*β* = 0.70, 95% CI: 0.24, 1.16, *P* < 0.001).

**Table 3:** Linear regression results of subgroup analysis by daily activity levels (Model 4)

Outcome variables			Activity level: low (*N* = 1257)			Activity level: medium (*N* = 863)			Activity level: high (*N* = 878)		
			β-Coefficients(95%CI)			β-Coefficients(95%CI)			β-Coefficients(95%CI)		
	Nighttime sleep duration	6–8 h	< 6 h	> 8 h		< 6 h	> 8 h		< 6 h	> 8 h	
Handgrip		Ref	-1.18(-2.15, -0.21)**	-0.74(-2.06, 0.59)		-0.91(-1.87, 0.05)	-1.23(-2.68, 0.22)		0.96(-0.01, 1.93)	0.48(-1.08, 2.05)	
ASM/HT^2^		Ref	0.01(-0.03, 0.05)	0.01(-0.04, 0.06)		0.00(-0.04, 0.03)	-0.02(-0.07, 0.03)		-0.01(-0.05, 0.03)	-0.04(-0.11, 0.02)	
Chair stand test		Ref	0.41(0.05, 0.77)*	0.60(0.11, 1.09)*		0.23(-0.32, 0.78)	-0.58(-1.42, 0.25)		-0.29(-0.99, 0.41)	0.26(-0.86, 1.38)	
Pace of walking		Ref	0.10(-0.58, 0.78)	1.30(0.37, 2.23)***		0.12(-0.04, 0.28)	0.23(-0.02, 0.47)		-0.16(-0.61, 0.28)	-0.09(-0.81, 0.63)	
SPPB score		Ref	-0.04(-0.24, 0.17)	-0.31(-0.59, -0.03)*		0.07(-0.18, 0.32)	-0.28(-0.66, 0.10)		-0.10(-0.36, 0.16)	0.19(-0.23, 0.61)	
	Variations in nighttime sleep duration	Not changed	Increased	Decreased		Increased	Decreased		Increased	Decreased	
Handgrip		Ref	-0.58(-1.48, 0.31)	0.30(-0.64, 1.24)		-1.23(-2.35, -0.11)*	-0.76(-1.92, 0.39)		0.05(-1.25, 1.34)	0.62(-0.64, 1.87)	
ASM/HT^2^		Ref	-0.02(-0.06, 0.02)	-0.01(-0.06, 0.03)		0.01(-0.03, 0.05)	-0.02(-0.06, 0.02)		0.04(-0.01, 0.09)	0.00(-0.05, 0.06)	
Chair stand test		Ref	0.29(-0.10, 0.67)	0.22(-0.18, 0.63)		0.05(-0.59, 0.70)	0.53(-0.13, 1.19)		0.45(-0.48, 1.38)	0.49(-0.41, 1.40)	
Pace of walking		Ref	0.46(-0.27, 1.19)	-0.08(-0.85, 0.69)		0.15(-0.04, 0.34)	0.04(-0.15, 0.23)		-0.26(-0.85, 0.34)	-0.55(-1.12, 0.03)	
SPPB score		Ref	-0.05(-0.27, 0.17)	-0.05(-0.28, 0.18)		-0.24(-0.54, 0.05)	-0.24(-0.54, 0.06)		-0.05(-0.41, 0.30)	-0.14(-0.48, 0.20)	
	Napping duration	No napping	< 30 min	30–90 min	> 90 min	< 30 min	30–90 min	> 90 min	< 30 min	30–90 min	> 90 min
Handgrip		Ref	1.24(-0.27, 2.76)	0.13(-0.69, 0.94)	-1.18(-2.25, -0.12)*	-0.19(-1.96, 1.59)	0.62(-0.36, 1.61)	-1.17(-2.52, 0.19)	1.53(-0.38, 3.44)	0.67(-0.38, 1.71)	0.20(-1.16, 1.56)
ASM/HT^2^		Ref	-0.02(-0.09, 0.04)	0.02(-0.01, 0.06)	0.03(-0.01, 0.08)	0.02(-0.05, 0.08)	0.01(-0.03, 0.04)	0.00(-0.05, 0.05)	-0.03(-0.11, 0.05)	-0.03(-0.07, 0.01)	-0.02(-0.08, 0.03)
Chair stand test		Ref	0.49(-0.16, 1.14)	0.20(-0.15, 0.55)	0.70(0.24, 1.16)***	-0.04(-1.06, 0.97)	0.46(-0.11, 1.02)	0.46(-0.32, 1.24)	-0.19(-1.56, 1.18)	0.03(-0.72, 0.79)	0.47(-0.51, 1.45)
Pace of walking		Ref	-0.44(-1.69, 0.80)	-0.37(-1.04, 0.30)	-0.20(-1.07, 0.68)	0.16(-0.14, 0.45)	-0.11(-0.27, 0.06)	-0.04(-0.27, 0.18)	-0.13(-1.01, 0.75)	0.06(-0.42, 0.54)	-0.03(-0.66, 0.59)
SPPB score		Ref	0.21(-0.16, 0.58)	-0.01(-0.21, 0.19)	-0.06(-0.33, 0.20)	-0.29(-0.75, 0.17)	0.26(0.00, 0.51)*	-0.06(-0.41, 0.29)	0.49(-0.03, 1.01)	0.06(-0.23, 0.34)	-0.03(-0.39, 0.34)

### Differences in the impact of sleep characteristics on physical functioning by gender

To explore age-related variations, we performed a subgroup analysis, dividing participants into an older group (> 65 years) and a younger group (≤ 65 years) (Table 4). In the older group, excessive napping (> 90 min) was associated with lower handgrip strength (*β* = -0.89, 95% CI: -1.69, -0.08, *P* < 0.05) and longer chair stand test time (*β* = 0.77, 95% CI: 0.30, 1.25, *P* < 0.01). Changes in nighttime sleep duration, whether an increase (*β* = 0.45, 95% CI: 0.04, 0.86, *P* < 0.05) or a decrease (*β* = 0.54, 95% CI: 0.12, 0.96, *P* < 0.05), were associated with longer chair stand test time. Moreover, increased nighttime sleep duration was negatively associated with handgrip strength (*β* = -0.74, 95% CI: -1.44, -0.04, *P* < 0.05). In contrast, among the younger group, only prolonged nighttime sleep (> 8 h) was significantly associated with slower walking speed (*β* = 3.06, 95% CI: 0.96, 5.16, *P* < 0.01), indicating fewer sleep-related effects on other physical function measures in this subgroup.

**Table 4:** Linear regression results of subgroup analysis by age groups (Model 4)

Outcome variables			Age < = 65 (*N* = 624)			Age > 65 (*N* = 2374)		
			β-Coefficients (95%CI)			β-Coefficients (95%CI)		
	Nighttime sleep duration	6–8 h	< 6 h	> 8 h		< 6 h	> 8 h	
Handgrip		Ref	-0.52(-1.74, 0.70)	-0.46(-2.43, 1.50)		-0.06(-0.66, 0.54)	-0.63(-1.50, 0.24)	
ASM/HT^2^		Ref	0.01(-0.04, 0.06)	0.04(-0.04, 0.12)		-0.01(-0.03, 0.02)	-0.03(-0.07, 0.00)	
Chair stand test		Ref	0.32(-0.23, 0.88)	-0.25(-1.14, 0.65)		0.07(-0.28, 0.42)	0.38(-0.13, 0.89)	
Pace of walking		Ref	-0.14(-1.44, 1.17)	3.06(0.96, 5.16)**		0.03(-0.16, 0.22)	0.14(-0.13, 0.41)	
SPPB score		Ref	0.23(-0.04, 0.51)	-0.02(-0.47, 0.42)		-0.09(-0.24, 0.07)	-0.20(-0.43, 0.02)	
	Variations in nighttime sleep duration	Not changed	Increased	Decreased		Increased	Decreased	
Handgrip		Ref	-0.42(-1.85, 1.01)	0.46(-0.96, 1.88)		-0.74(-1.44, -0.04)*	-0.05(-0.77, 0.67)	
ASM/HT^2^		Ref	0.02(-0.04, 0.08)	-0.04(-0.09, 0.02)		0.00(-0.03, 0.03)	-0.01(-0.04, 0.02)	
Chair stand test		Ref	-0.16(-0.81, 0.50)	-0.13(-0.78, 0.52)		0.45(0.04, 0.86)*	0.54(0.12, 0.96)*	
Pace of walking		Ref	0.84(-0.70, 2.38)	-0.17(-1.70, 1.36)		0.05(-0.17, 0.27)	-0.17(-0.40, 0.05)	
SPPB score		Ref	-0.14(-0.47, 0.18)	-0.22(-0.54, 0.10)		-0.11(-0.29, 0.08)	-0.09(-0.28, 0.09)	
	Napping duration	No napping	< 30 min	30–90 min	> 90 min	< 30 min	30–90 min	> 90 min
Handgrip		Ref	1.36(-0.93, 3.65)	-0.39(-1.63, 0.85)	-0.01(-1.72, 1.69)	0.73(-0.40, 1.85)	0.60(-0.02, 1.21)	-0.89(-1.69, -0.08)*
ASM/HT^2^		Ref	-0.06(-0.16, 0.04)	0.01(-0.04, 0.07)	0.04(-0.04, 0.11)	0.00(-0.04, 0.05)	0.00(-0.02, 0.03)	0.00(-0.03, 0.04)
Chair stand test		Ref	-0.27(-1.31, 0.77)	0.32(-0.25, 0.88)	-0.24(-1.02, 0.54)	0.26(-0.40, 0.92)	0.23(-0.14, 0.59)	0.77(0.30, 1.25)**
Pace of walking		Ref	-0.81(-3.28, 1.65)	-0.56(-1.89, 0.77)	-0.66(-2.50, 1.18)	0.03(-0.32, 0.38)	-0.02(-0.21, 0.17)	0.08(-0.18, 0.33)
SPPB score		Ref	0.02(-0.50, 0.55)	-0.03(-0.32, 0.25)	-0.17(-0.56, 0.22)	0.17(-0.13, 0.46)	0.10(-0.06, 0.26)	-0.03(-0.24, 0.17)

### Gender differences in the impact of sleep characteristics on physical functioning

The impact of sleep characteristics on physical function varied significantly between males and females (Table 5). In males, both moderate (30–90 min) and excessive (> 90 min) napping were associated with longer chair stand test time (*β* = 0.53, 95% CI: 0.18, 0.89, *P* < 0.001; *β* = 0.66, 95% CI: 0.21, 1.11, *P* < 0.001). Additionally, reduced nighttime sleep duration was correlated with prolonged chair stand test time (*β* = 0.46, 95% CI: 0.05, 0.87, *P* < 0.05). Increased nighttime sleep duration was also negatively associated with handgrip strength (*β* = -0.96, 95% CI: -1.80, -0.12, *P* < 0.05), whereas decreased nighttime sleep duration was linked to lower SPPB scores (*β* = -0.21, 95% CI: -0.42, -0.01, *P* < 0.05). Conversely, in females, the primary significant association observed was between prolonged nighttime sleep (> 8 h) and slower walking speed (*β* = 1.32, 95% CI: 0.31, 2.34, *P* < 0.01), suggesting fewer adverse sleep-related effects on other physical function measures compared to males.

**Table 5:** Linear regression results of subgroup analysis by gender (Model 4)

Outcome variables			Female (*N* = 1362)			Male (*N* = 1636)		
			β-Coefficients (95%CI)			β-Coefficients (95%CI)		
	Nighttime sleep duration	6–8 h	< 6 h	> 8 h		< 6 h	> 8 h	
Handgrip		Ref	-0.32(-1.00, 0.36)	-0.95(-2.03, 0.14)		0.17(-0.61, 0.96)	-0.12(-1.20, 0.95)	
ASM/HT^2^		Ref	-5.00(-0.03, 0.03)	-2.00(-0.08, 0.03)		-0.01(-0.03, 0.02)	-0.01(-0.05, 0.03)	
Chair stand test		Ref	0.12(-0.37, 0.60)	-0.02(-0.80, 0.76)		0.13(-0.24, 0.49)	0.37(-0.13, 0.87)	
Pace of walking		Ref	-0.05(-0.69, 0.58)	1.32(0.31, 2.34)**		0.06(-0.07, 0.19)	0.09(-0.09, 0.28)	
SPPB score		Ref	0.08(-0.12, 0.29)	-0.23(-0.56, 0.10)		-0.12(-0.30, 0.06)	-0.12(-0.37, 0.13)	
	Variations in nighttime sleep duration	Not changed	Increased	Decreased		Increased	Decreased	
Handgrip		Ref	0.15(-0.73, 1.03)	0.77(-0.10, 1.63)		-0.96(-1.80, -0.12)*	-0.34(-1.22, 0.53)	
ASM/HT^2^		Ref	-0.01(-0.05, 0.03)	-0.05(-0.09, 0.00)		0.02(-0.01, 0.05)	0.01(-0.02, 0.05)	
Chair stand test		Ref	0.14(-0.49, 0.77)	0.30(-0.33, 0.92)		0.38(-0.01, 0.78)	0.46(0.05, 0.87)*	
Pace of walking		Ref	0.27(-0.55, 1.09)	-0.28(-1.09, 0.53)		0.14(-0.01, 0.28)	-0.07(-0.22, 0.08)	
SPPB score		Ref	-0.09(-0.36, 0.18)	-0.02(-0.28, 0.24)		-0.12(-0.32, 0.07)	-0.21(-0.42, -0.01)*	
	Napping duration	No napping	< 30 min	30–90 min	> 90 min	< 30 min	30–90 min	> 90 min
Handgrip		Ref	0.76(-0.49, 2.01)	0.59(-0.13, 1.30)	-0.94(-1.98, 0.10)	0.97(-0.52, 2.46)	0.32(-0.45, 1.10)	-0.60(-1.56, 0.36)
ASM/HT^2^		Ref	-0.01(-0.07, 0.05)	0.01(-0.03, 0.04)	0.02(-0.04, 0.07)	-0.02(-0.07, -0.03)	0.01(-0.02, 0.03)	0.01(-0.03, 0.04)
Chair stand test		Ref	-0.13(-1.02, 0.76)	-0.08(-0.60, 0.43)	0.59(-0.15, 1.34)	0.40(-0.29, 1.09)	0.53(0.18, 0.89)***	0.66(0.21, 1.11)***
Pace of walking		Ref	-0.38(-1.55, 0.78)	-0.30(-0.97, 0.37)	-0.40(-1.37, 0.57)	0.05(-0.21, 0.30)	-0.01(-0.14, 0.13)	0.13(-0.04, 0.29)
SPPB score		Ref	-0.01(-0.38, 0.37)	0.03(-0.18, 0.25)	-0.18(-0.49, 0.13)	0.30(-0.04, 0.65)	0.11(-0.07, 0.29)	0.03(-0.19, 0.25)

### The combined effect of nighttime sleep and napping

Table 6 illustrated the combined effect of nighttime sleep and napping. Compared to individuals with less than 6 h of nighttime sleep and no napping, those who slept 6–8 h at night and napped for 30–90 min had longer chair stand test times (*β* = 0.63, 95% CI: 0.06–1.20, *P* < 0.05). Similarly, individuals with 6–8 h of nighttime sleep and naps exceeding 90 min showed longer walking times (*β* = 1.44, 95% CI: 0.68–2.21, *P* < 0.01). This suggests that while moderate napping combined with sufficient nighttime sleep may support physical function, excessive napping could be associated with slower mobility, highlighting the importance of balanced sleep patterns for maintaining optimal physical performance in middle-aged and elderly individuals.

**Table 6:** The combined effect of nighttime sleep and napping duration on outcomes (Model 4)

Nighttime sleep duration	Napping duration	Case/*N*(%)	Handgrip (β-Coefficients (95%CI))	ASM/HT^2^ (β-Coefficients (95%CI))	Chair stand test (β-Coefficients (95%CI))	Pace of walking (β-Coefficients (95%CI))	SPPB score (β-Coefficients (95%CI))
< 6 h	No snaps	453/15.11%	Ref	Ref	Ref	Ref	Ref
	< 30 min	59/1.97%	1.19(-0.59,2.97)	0.03(-0.05,0.10)	-0.02(-1.03,0.99)	-0.25(-1.29,0.80)	0.27(-0.19,0.73)
	30–90 min	363/12.11%	0.63(-0.27,1.54)	0.01(-0.02,0.05)	0.18(-0.34,0.70)	-0.18(-0.71,0.35)	0.18(-0.06,0.41)
	> 90 min	125/4.17%	-0.85(-2.15,0.46)	0.04(-0.01,0.09)	-0.08(-0.82,0.67)	0.04(-0.72,0.81)	-0.11(-0.45,0.23)
6–8 h	No napping	610/20.35%	0.21(-0.60,1.02)	0.02(-0.01,0.05)	-0.36(-0.82,0.10)	-0.13(-0.61,0.34)	0.08(-0.13,0.29)
	< 30 min	118/3.94%	1.15(-0.18,2.49)	-0.02(-0.07,0.03)	-0.15(-0.91,0.61)	-0.08(-0.87,0.70)	0.19(-0.16,0.53)
	30–90 min	665/22.18%	0.60(-0.20,1.39)	0.01(-0.02,0.04)	-0.04(-0.50,0.41)	-0.01(-0.48,0.46)	0.09(-0.12,0.29)
	> 90 min	271/9.04%	-0.64(-1.64,0.36)	0.02(-0.02,0.06)	0.63(0.06,1.20)*	-0.01(-0.60,0.57)	0.05(-0.20,0.31)
> 8 h	No snaps	126/4.20%	0.03(-1.27,1.33)	-0.02(-0.07,0.03)	-0.09(-0.83,0.65)	1.44(0.68,2.21)***	-0.11(-0.44,0.23)
	< 30 min	23/0.77%	-0.31(-3.06,2.45)	0.01(-0.11,0.12)	0.23(-1.32,1.79)	0.30(-1.31,1.92)	-0.17(-0.88,0.54)
	30–90 min	121/4.04%	-0.40(-1.72,0.93)	0.03(-0.02,0.08)	0.30(-0.45,1.05)	-0.00(-0.78,0.77)	0.03(-0.31,0.37)
	> 90 min	64/2.13%	0.00(-1.72,1.73)	-0.03(-0.10,0.04)	0.29(-0.69,1.27)	-0.06(-1.07,0.96)	-0.21(-0.65,0.24)

## Discussion

This study found that both excessively short and long nighttime sleep durations, as well as longer napping durations, can negatively affect physical functioning in older adults. Specifically, napping for more than 90 min was linked to decreased handgrip strength (*β* = -0.39, 95% CI: -1.43, -0.01, *P* < 0.05) and prolonged chair stand test time (*β* = 0.59, 95% CI: 0.18, 0.99, *P* < 0.01). RCS indicated that an potentially optimal napping duration of around 50 min was beneficial for maintaining handgrip strength. Additionally, the joint analysis revealed that individuals with moderate nighttime sleep duration but longer naps performed worse on physical tests. Subgroup analysis highlighted that maintaining healthy sleep habits was particularly important for men over 65 with low daily activity levels. These findings underscore the complex relationship between sleep patterns and physical functioning in older adults, suggesting that both adequate nighttime sleep and appropriate napping duration are crucial for preserving physical function in this population.

In our cohort, the average baseline nighttime sleep duration for middle-aged and older adults was 6.32 ± 2.10 h, indicating that the nighttime sleep duration for Chinese middle-aged and older adults is significantly below the recommendation of the National Sleep Foundation [19]. Insufficient sleep is associated with a range of adverse health outcomes, including increased risk of chronic conditions such as cardiovascular disease, diabetes, and mental health disorders. Our study confirms that interventions on sleep characteristics in middle-aged and older adults may affect their physical functioning. In the subgroup analyses controlling for confounders, we observe associations between nighttime sleep duration and certain physical functioning measures that are not evident in the overall linear models. This discrepancy may be attributable to the heterogeneity within the general population model. Although these findings require cautious interpretation, they are consistent with some existing studies [8, 9]. Both excessively short and long nighttime sleep durations have been shown to adversely affect physical function, underscoring the importance of maintaining an optimal sleep duration. Several mechanisms may explain this relationship. Disruptions in circadian rhythm, which regulate essential physiological processes, can impair muscle repair and metabolism. For instance, insufficient sleep or irregular sleep patterns may lead to disturbances in the secretion of growth hormone, which is critical for muscle tissue regeneration [20]. Abnormal sleep patterns can influence blood glucose regulation, blood pressure, cognitive function, and hormonal balance (especially as cortisol and melatonin), all of which are essential for muscle protein synthesis and breakdown [21, 22]. Short sleep duration has been linked to insulin resistance and impaired glucose metabolism, which in turn may reduce energy availability for muscle function and repair. Elevated cortisol levels, often seen with sleep deprivation or excessive sleep, may promote muscle catabolism (breakdown) and inhibit muscle growth. Yang H [23] established mouse models of sleep deprivation and observed that in sleep-deprived mice led to a significant decline in limb grip strength, a reduction in gastrocnemius muscle mass and muscle index, as well as a marked decrease in the melatonin to cortisol ratio, while exposure to arsenite. Some studies have also suggested that the relationship between sleep duration and muscle mass and function may be linked to testosterone secretion, which could explain the stronger associations observed in the male subgroup. Some studies have also suggested that the relationship between sleep duration and muscle mass and function may be related to the secretion of testosterone [24]. A multicenter clinical study conducted in Europe showed that among middle-aged and elderly individuals aged 30 to 79, average testosterone levels declined as sleep quality deteriorated [25]. We observed that the higher number of positive results in the male subgroup may also be related to this factor. Regarding extended nighttime sleep duration, some studies have indicated that extreme long sleep duration is associated with higher levels of CRP (ES 0.17; 95% CI 0.01–0.34) and IL-6 (ES 0.11; 95% CI 0.02–0.20) [26]. Some observational studies have also noted a strong association between excessively long sleep duration (generally considered to be over 9 h) and sarcopenia [5, 27]. Our study suggests that an appropriate duration of nighttime sleep may have a protective effect on the physical function of middle-aged and elderly individuals.

Our subgroup analysis revealed that the impact of abnormal sleep patterns on physical functioning was more pronounced in individuals with low daily activity levels, older adults, and males. The differences between the overall and subgroup analyses may stem from population heterogeneity. In the general analysis, the diverse characteristics of the population may obscure certain associations due to variations in baseline physical function, lifestyle factors, and physiological resilience. However, within more homogeneous subgroups sharing similar risk factors, the detrimental effects of sleep disturbances on physical function become more evident, highlighting patterns that might not be detectable in the broader population. Our hypothesis is as follows: In individuals with lower daily activity levels, the impact of insufficient or excessive sleep may be more pronounced due to their already diminished baseline physical function. Physical inactivity itself is a well-established risk factor for muscle weakness, and disruptions in sleep patterns could further exacerbate these issues [28]. Among older adults (aged over 65), who are inherently at a higher risk of frailty, abnormal sleep patterns may have a more substantial impact. This population is also more likely to experience cognitive decline, which can further impair motor function and balance [29]. In males, the association between sleep disturbances and physical function may be influenced by hormonal factors, particularly testosterone. Testosterone plays a crucial role in maintaining muscle mass and strength, and disruptions in sleep—especially those that alter hormonal balance—may have a more pronounced effect on physical performance in men [24, 25]. Additionally, given that males generally have greater muscle mass, they may be more sensitive to the detrimental effects of sleep disturbances on muscle strength and overall physical function. We believe that these subgroups represent high-risk populations for functional decline, making them key targets for potential interventions aimed at mitigating the adverse effects of sleep disturbances on physical health. Identifying and addressing sleep-related risk factors in these vulnerable groups could help reduce the burden of age-related physical decline, promote independent living in these groups, and alleviate healthcare costs associated with mobility impairments and frailty-related complications.

Currently, there is limited research on the association between daytime napping and health outcomes. Some research showed that participants who napped for over 90 min per day have higher prevalence rates of type 2 diabetes and obesity compared to those who nap for 5 to 30 min per day [30–32]. In contrast, another research based on CHARLS found that older adults who napped for less than 30 min exhibited a higher risk of frailty, whereas those who napped for a moderate duration (more than 30 min) exhibited the opposite result [32]. Our linear regression analysis of the overall population revealed a U-shaped relationship between nap duration and handgrip strength, with data suggesting that around 50 min of napping may be associated with potentially optimal handgrip strength. However, the determination of a healthy napping duration remains contentious, primarily due to the diverse nature of napping characteristics. Among middle-aged and older adults, the reasons for napping differ: some nap to compensate for inadequate nighttime sleep, others nap out of boredom, and some have a habitual napping routine regardless of their nighttime sleep duration [32–35]. In our study, we carefully controlled for potential confounding factors, including daily activity levels in subgroup analyses. The results indicated that napping for more than 90 min was associated with a decline in handgrip strength (*β* = -1.18, 95% CI: -2.25, -0.12, *P* < 0.05) and longer chair stand test time (*β* = 0.70, 95% CI: 0.24, 1.16, *P* < 0.001). Interestingly, within the range of nighttime sleep duration between 6 and 8 h, longer nap durations appeared to be associated with worse physical function. This also suggested that prolonged, low-energy napping might pose a risk to the physical functioning of older adults. Previous studies had reported the association between nap duration and sarcopenia among older Chinese adults: They found that individuals who napped for 1–59 min and 60–119 min had a significantly reduced risk of sarcopenia [36]. Additionally, research on athletes has shown that both short and long-term napping habits are associated with lower levels of muscle damage and oxidative stress markers compared to individuals who do not nap [37]. Some scholars noted that daytime napping can mitigate the microvascular immune-inflammatory effects caused by nighttime sleep deprivation and improve oxidative stress [38–40]. Nevertheless, further research is needed to better understand the complex impact of napping characteristics on the health of middle-aged and elderly individuals.

The distribution of sleep duration is shaped by a range of demographic, lifestyle, and health-related factors, making the relationship between nighttime sleep and daytime napping particularly complex. Our study is among the few to comprehensively examine the association between overall sleep patterns and physical function in middle-aged and older adults. A key strength of our study lies in its use of a nationally representative cohort from across China, along with rigorous control of potential confounders. Notably, we account for daily activities as covariates and employe RCS to assess the dose-response relationship between sleep duration and physical function. Furthermore, by analyzing the combined effects of nighttime sleep and napping duration, our study provides a more nuanced understanding of how different sleep patterns collectively influence physical health. Given that the baseline nighttime sleep duration in our cohort is notably lower than the recommendations of the National Sleep Foundation, these findings have important public health implications. Insufficient sleep is a growing concern among aging populations, with potential consequences for functional decline, frailty, and overall quality of life. Understanding how sleep patterns interact with physical function is crucial for developing targeted interventions to promote healthy aging. By highlighting the significance of balanced sleep habits, our findings may inform future public health strategies aimed at mitigating the risks associated with abnormal sleep patterns in older adults. These methodological approaches strengthen the credibility of our study and offer valuable insights for guiding future research and healthcare policies to improve the well-being of aging populations.

This study has several limitations. First, the assessment of participants’ sleep duration was primarily based on self-reported questionnaires. Despite excluding participants with mental or memory-related disorders, the findings may still be subject to recall bias and reporting inaccuracies, potentially leading to misclassification [41]. Second, our study only considered sleep duration and lacked an evaluation of sleep quality, such as sleep fragmentation, early awakenings, or night disturbances. Additionally, participants with poorer physical function were less likely to complete the physical performance tests, and the exclusion of these missing values may have introduced selection bias. We estimated participants’ muscle mass using anthropometric equations rather than Dual-Energy X-ray absorptiometry. While the formula has proven highly effective in the Chinese population, caution should be exercised when generalizing these findings to other ethnic groups [17, 42]. Furthermore, while our study identified an optimal nap duration for maintaining grip strength, this recommendation is based on observational data rather than a definitive causal conclusion, and individual differences in sleep needs should be considered. Finally, as a cohort study with a follow-up design, our findings provide valuable insights but do not establish causality. Future research should incorporate randomized controlled trials to further validate these associations and explore potential mechanisms underlying the observed relationships.

## Conclusions

Our study demonstrates that maintaining an adequate and stable sleep duration benefits the physical function of older adults. Both excessively short and long nighttime sleep, as well as extended daytime napping, were linked to poorer physical performance. These findings highlight the need for tailored sleep recommendations, particularly for high-risk groups such as older individuals, males, and those with low activity levels. Given the aging population and rising sleep disturbances, our results underscore the importance of incorporating sleep health into public health strategies to support mobility and independence in older adults.

## Electronic supplementary material

Below is the link to the electronic supplementary material.

Supplementary Material 1

## Data Availability

The original data for this study can be obtained by applying through the CHARLS (https://charls.pku.edu.cn/en/). The processed dataset from the current study is available upon reasonable request from the corresponding author.

## Abbreviations

CHARLS: China Health and Retirement Longitudinal Study
SPPB: Short Physical Performance Battery
AWGS: Asian Working Group for Sarcopenia
ASM: Appendicular skeletal muscle
RCS: Restricted cubic spline
