# Supplementary material for: Association between sleep characteristics and physical functioning in middle-aged and elderly adults: findings from Chinese cohorts
Source: Aging Clin Exp Res. 2025 Apr 7;37(1):118. doi: 10.1007/s40520-025-03020-9 (PMC11976827; doi:10.1007/s40520-025-03020-9)
Supplement: Supplementary file 1 — Supplementary Material 1 [file 40520_2025_3020_MOESM1_ESM.docx]

**1.SPPB score:**

1. Standing balance:

Instruct participants to stand in three different stances: SEMI-TANDEM, FULL-TANDEM, and SIDE-BY-SIDE, and record the duration they could maintain each stance.

1. Walking speed:

Instruct participants to walk along a 2.5-meter distance at their normal pace twice, and record the average speed taken for both walks.

1. Chair stand test:

| The SPPB score equals the sum of the scores of the above three tests, with higher scores indicating better overall physical functioning of the subject. |  |
| --- | --- |
| **Testing protocol** | **Score** |
| 1. Standing test |  |
| Maintain a semi-tandem stance for a continuous period of 10 seconds = TRUE | 1 |
| Maintain a stance with side by side for 10 seconds = TRUE | 2 |
| Maintain a full stance for a continuous period of 30 seconds (for individuals aged 70 and above) or 60 seconds (for individuals aged below 70) = TRUE | 4 |
| 2. Walking speed |  |
| Walking speed < 0.6m/s | 0 |
| Walking speed = 0.6 ~ 0.79m/s | 1 |
| Walking speed = 0.8 ~ 0.99m/s | 2 |
| Walking speed = 1.0 ~ 1.99m/s | 3 |
| Walking speed > 1.2m/s | 4 |
| 3. Chair stand test |  |
| Chair stand test not completed = TRUE | 0 |
| Chair stand time > 16.9s | 1 |
| Chair stand time = 13.7 ~ 16.9s | 2 |
| Chair stand time = 11.2 ~ 13.6s | 3 |
| Chair stand time < 11.2s | 4 |
|  |  |
|  |  |
|  |  |
|  |  |

Instruct the participants to perform the chair stand test five times consecutively, and record the average time taken to complete the task.

**2.** **Details about physical functioning measurement**

Each participant's dominant handgrip strength was measured using the TMWL-1000 dynamometer (Manufactured by Nantong Yuejian Physical Measurement Instrument). Each participant underwent two tests, and the average of these two measurements was recorded as their handgrip strength result. For the chair stand test, participants were required to stand up five times consecutively from a seated position with their arms crossed over their chest, and the time taken to complete this task was recorded. The walking test required participants to walk at their normal pace over a distance of 2.5 meters, repeated twice. The average time taken for the two trials was recorded as the result. ASM in Chinese individuals is calculated using the following formula: “ASM = 0.107 × height (cm) + 0.193 × weight (kg) − 0.037 × age − 4.157 × gender − 2.631”, with gender coded as 0 for females and 1 for males. ASM/HT^2^ based on individual height was used as a tool to evaluate muscle mass.

**3.BMI Calculation**

BMI was calculated as weight (kg) divided by the square of height (m²).

Weight Measurement: Participants’ body weight was measured using a calibrated scale. They were required to remove their shoes and stand upright on the scale while trained personnel recorded the readings.

Height Measurement: Height was measured using a stadiometer. Participants stood barefoot with their heels together and toes pointing outward at approximately 60°, maintaining an upright posture with their back against the vertical board of the stadiometer. Arms were relaxed at the sides, and the head was positioned in the Frankfort horizontal plane. Trained personnel recorded the height measurements.

**4. Comorbidity Assessment:**

Comorbid conditions were assessed using a structured questionnaire. Participants were asked the following question: "Have you been diagnosed with [conditions listed below, read one by one] by a doctor?"

The questionnaire included 14 common chronic diseases, which were read aloud to participants to ensure accurate self-reporting.

Based on the number of reported comorbidities, participants’ general health status was classified into four categories:

1.No comorbidities

2.One comorbidity

3.Two comorbidities

4.Three or more comorbidities

**5.Questionnaire about the presence of 14 common comorbidities:**

Question: Have you been diagnosed with [conditions listed below, read one by one] by a doctor?

1. Hypertension

2. Dyslipidemia (elevation of low density lipoprotein, triglycerides (TGs),and total

cholesterol, or a low high density lipoprotein level)

3. Diabetes or high blood sugar

4. Cancer or malignant tumor (excluding minor skin cancers)

5. Chronic lung diseases, such as chronic bronchitis , emphysema ( excluding tumors, or cancer)

6. Liver disease (except fatty liver, tumors, and cancer)

7. Heart attack, coronary heart disease, angina, congestive heart failure, or other heart problems

8. Stroke

9. Kidney disease (except for tumor or cancer)

10. Stomach or other digestive disease (except for tumor or cancer)

11. Emotional, nervous, or psychiatric problems

12. Memory-related disease

13. Arthritis or rheumatism

14. Asthma

**6. Daily Activity Score Assessment:**

Daily activity levels were assessed using a retrospective questionnaire, in which participants recalled their ability to perform general daily activities.

The questionnaire included tasks such as walking on level ground, climbing stairs, bending, squatting, and carrying heavy objects.

Based on the responses, participants were categorized into three activity levels (low, medium, and high) according to tertiles of the total activity score.

**7.Daily activity scores**

|  |  |
| --- | --- |
| **Question protocol** | **Score** |
| **1. Flat ground activities** |  |
| Have no difficulty with running or jogging about 1 Km | 3 |
| Have no difficulty with walking 1 km | 2 |
| Have no difficulty with walking 100 metres | 1 |
| None of the above activities could be completed independently | 0 |
| **2. Do you have difficulty with getting up from a chair after sitting for a long period?** |  |
| No, I don’t have any difficulty | 3 |
| I have difficulty but can still do it | 2 |
| Yes, I have difficulty and need help | 1 |
| I can not do it | 0 |
| **3. Do you have difficulty with climbing several flights of stairs without resting?** |  |
| No, I don’t have any difficulty | 3 |
| I have difficulty but can still do it | 2 |
| Yes, I have difficulty and need help | 1 |
| I can not do it | 0 |
| **4. Do you have difficulty with stooping, kneeling, or crouching?** |  |
| No, I don’t have any difficulty | 3 |
| I have difficulty but can still do it | 2 |
| Yes, I have difficulty and need help | 1 |
| I can not do it | 0 |
| **5. Do you have difficulty with reaching or extending your arms above shoulder level?  (he/she is regarded as not having difficulty only if he/she can extend both of his/her  arms, otherwise he/she is regarded as having difficulty.)** |  |
| No, I don’t have any difficulty | 3 |
| I have difficulty but can still do it | 2 |
| Yes, I have difficulty and need help | 1 |
| I can not do it | 0 |
| **6. Do you have difficulty with lifting or carrying weights over 10 jin(5 kg), like a heavy bag  of groceries?** |  |
| No, I don’t have any difficulty | 3 |
| I have difficulty but can still do it | 2 |
| Yes, I have difficulty and need help | 1 |
| I can not do it | 0 |
| **7. Do you have difficulty with picking up a small coin from a table?** |  |
| No, I don’t have any difficulty | 3 |
| I have difficulty but can still do it | 2 |
| Yes, I have difficulty and need help | 1 |
| I can not do it | 0 |
|  |  |
